# Influence of Polymorphism on Glycosylation of Serum Amyloid A4 Protein

**DOI:** 10.1155/2014/527254

**Published:** 2014-05-15

**Authors:** Toshiyuki Yamada, Jyunji Sato, Kazuhiko Kotani, Masafumi Tanaka

**Affiliations:** ^1^Department of Clinical Laboratory Medicine, Jichi Medical University, Tochigi 329-0498, Japan; ^2^Department of Biophysical Chemistry, Kobe Pharmaceutical University, Hyogo 658-8558, Japan

## Abstract

Serum amyloid A4 (SAA4) is a constitutive apolipoprotein of high-density lipoprotein. It exhibits N-linked glycosylation in its second half. There are both glycosylated and nonglycosylated forms in plasma and the ratio of these two forms varies among individuals. This study was conducted to examine the influence of genetic polymorphism of SAA4 on its glycosylation status. In 55 healthy subjects, SAA4 polymorphism was analyzed by PCR combined direct sequencing and its glycosylation status was analyzed by immunoblotting. The results showed that the percentage of glycosylation in subjects with amino acid substitutions at positions 71 and/or 84 was significantly (*P* < 0.05) higher than that in subjects with the wild type. The polymorphism had no influence on the plasma concentration of SAA4. These findings suggest that the changes in protein structures alter the efficiency of glycosylation in the SAA4 molecule. The functional implication of this should be of interest.

## 1. Introduction

**Figure 1:**
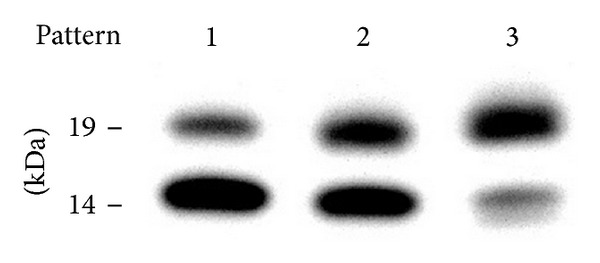
Representative immunoblotting patterns for SAA4 in plasma. Each sample of patterns 1, 2, and 3 is from a subject showing SAA4 genotype as the wild type, a substitution at position 71, and substitutions at positions 71 and 84, respectively. Estimated molecular weight is shown.

Serum amyloid A (SAA) is a polymorphic protein [1–4]. In humans, SAA proteins are coded at four loci, called* SAA1*,* SAA2*,* SAA3*, and* SAA4*.* SAA3* is a pseudogene, while the others encode products, which are screted from the liver and bound to high-density lipoprotein (HDL) in the blood. The synthesis of SAA1 and SAA2 (acute phase SAA; A-SAA) is increased in inflammatory disorders. They may play roles in the immune system, lipoprotein metabolism, and tissue repair during or subsequent to inflammation [2, 3]. They are also serum precursors of AA proteins, the chief constituents of reactive amyloid deposits [4]. Unlike A-SAA, SAA4, constitutive SAA in other words, is not markedly elevated in inflammation and does not form amyloid fibrils [5–7], except for its variant [8]. SAA4 has 112 amino acids, with an insertion of eight amino acids at the position corresponding to residue 70 of A-SAA [5]. This insertion generates N-linked glycosylation. Interestingly, SAA4 is not completely glycosylated; two forms, glycosylated (G) and nonglycosylated (NG), are observed in plasma (Figure 1). Since we noted that the ratio of G : NG varies among individuals and might be constant within individuals, this study aimed to examine whether the glycosylation of SAA4 is genetically regulated.

## 2. Materials and Methods

### 2.1. Samples

The study protocol was approved by the Bioethics Committee for Human Genome and Gene Analysis, Jichi Medical University (No. 13–17). Fifty-two healthy adults (22 females and 30 males), aged from 21 to 80, voluntarily participated in this study. Since a rare polymorphism was found at two sites in a female, her husband, son, and daughter were added to the analyses. Blood was drawn into a tube containing EDTA. After centrifugation, plasma was obtained and kept at −20°C and DNA was extracted from the buffy coat and stored at −20°C.

### 2.2. Immunoblotting

Plasma was diluted 1 : 100 with electrophoresis sample buffer and 10 *μ*L of it was subjected to tricine sodium dodecyl sulfate polyacrylamide gel electrophoresis followed by transfer to a polyvinyl difluoride membrane, as previously described [7]. The membrane was blocked in 1% bovine serum albumin (BSA) in phosphate-buffered saline (PBS) for 1 hour at room temperature (RT) and then reacted with 2 *μ*g/mL rabbit anti-human SAA4 antibodies [9] for 1 hour at RT, followed by reaction with 1 : 1000-diluted peroxidase-conjugated anti-rabbit immunoglobulins (Bioscience Inc., USA) for 1 hour at RT. Then, as a substrate, EzWestLumi plus (ATTO Corp., Japan) was applied and the output was analyzed using a chemiluminescent image analyzer system, Ez-Capture MG (ATTO). Using CS Analyzer 3.0 software in that system, the intensities of the two SAA4-corresponding bands were read. The SAA4 concentration of each sample was calculated from the intensities of the two bands by comparison with those of the serum, the SAA4 concentration of which was known [9].

### 2.3. DNA Analysis

SAA4 exons 2, 3, and 4 were subjected to PCR followed by direct sequencing as previously reported [8]. This was performed on the selected subjects as described below. On the basis of the SNPs found in exon 4, PCR-RFLP methods were established for the screening analysis. PCR was performed for 30 cycles of 94°C, 1 min; 54°C, 1 min; and 72°C, 1 min using a forward primer (CCAGGGTCTATCTTCAGGGATTAATAGAGT), which included a restriction site and a reverse primer (TTCAGTATTTCTTAGGCAGGCCGTCAGGTC). Digestion with AfaI (Takara, Japan), which cuts the wild-type sequence at residue 71, or TaqI (Takara), which cuts the wild-type sequence at residue 84, was evaluated on an agarose gel.

## 3. Results

Immunoblotting findings were largely divided into three patterns (Figure 1): pattern 1: nonglycosylated form (NG) dominant (corresponding to percentage of glycosylation <40% in Figure 3), pattern 3: glycosylated form (G) dominant (corresponding to percentage of glycosylation >60% in Figure 3), and pattern 2: intermediate between patterns 1 and 3. Out of 52 subjects, 42 (80.8%), 6 (11.5%), and 4 (7.7%) showed patterns 1, 2, and 3, respectively. A faint band was seen just below the nonglycosylated SAA4 of pattern 3. It may have corresponded to a posttranslationally C-terminus-degraded species [10], although this was not confirmed.

**Figure 2:**
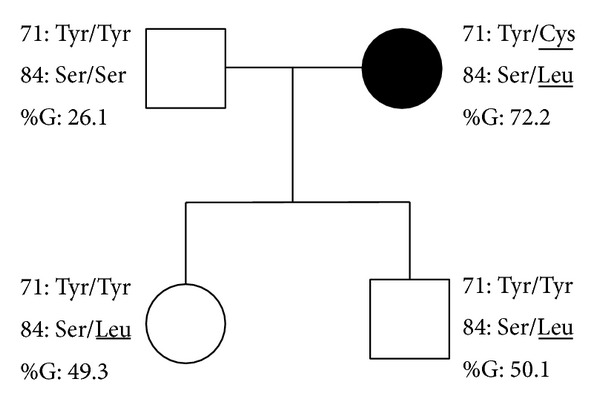
Family analysis of SAA4 polymorphism and the percentage of glycosylation (%G). Substituted amino acids are underlined. ●: proband showing two substitutions.

DNA sequencing of the four exons was performed for two subjects in each group showing the different patterns in immunoblotting. The data for subjects showing pattern 1 were in complete agreement with a previous report [5] and are referred to as the wild type here. Subjects showing pattern 2 had an allele with a substitution at residue 71 from TAC to TGC, which would change the amino acid from tyrosine to cysteine. One out of the two subjects showing pattern 3 had an allele with a substitution at residue 84 from TCG to TTG, which changes the amino acid from serine to leucine. The other had the same substitutions as above at residues 71 and 84. In order to define the haplotype of the last subject, her husband and two children were analyzed. The husband showed pattern 1 for immunoblotting and homozygosity for the wild-type sequence, while both children showed pattern 2 and heterozygosity for residue 84 substitution (Figure 2). Therefore, the proband showing two substitutions may have one allele with ^71^Tyr and ^84^Leu and the other with ^71^Cys and ^84^Ser.

**Figure 3:**
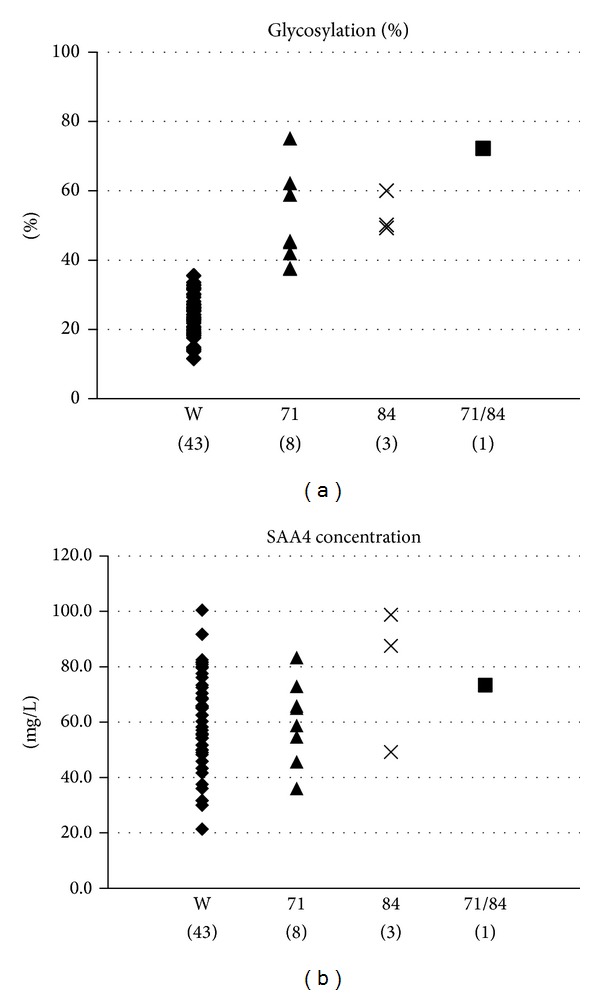
The percent glycosylation (%G) of SAA4 (a) and SAA4 concentration (b) by genotype. W: wild type, 71: with an allele with ^71^Cys, 84: with an allele with ^84^Leu, and 71/84: with both alleles. Number of subjects is shown in parentheses.

Since substitutions were not found in the other exons, the two SNPs in exon 4 were analyzed for the remaining subjects. The percentage of glycosylated form (%G) and plasma SAA4 concentrations was compared among the groups showing each polymorphism (Figure 3). The mean %G of subjects with wild type, those with a ^71^Tyr substitution, and those with a ^84^Ser substitution was 24.1%, 50.5%, and 53.1%, respectively. The %G with either substitution was significantly (*P* < 0.05) higher than that of the wild type. No difference in %G was noted between substitutions of ^71^Tyr and ^84^Ser. There was also no difference in SAA4 concentration among the groups.

## 4. Discussion

This study revealed that polymorphism of SAA4 influenced glycosylation efficiency. N-glycosylation is a phenomenon in which an oligosaccharide is linked to the asparagine side chain by oligosaccharyl transferase. The structural environment around asparagine may thus determine the glycosylation efficiency by altering the access of oligosaccharide or oligosaccharyl transferase. However, since the tertiary structure of SAA4 has not been elucidated yet, discussion cannot go beyond speculation. It has been postulated that the unfolding of polypeptide chains is required in order to expose appropriate asparagine sites for carbohydrate attachment [11]. Indeed, the computer-based secondary structure predictions (PSIPRED and GOR4 methods) indicate that the N-glycosylation site in the SAA4 molecule adopts a random coil conformation irrespective of the amino acid substitutions at residues 71 and 84 [12, 13]. Conformational differences induced by the amino acid substitutions existed only in the possible helical regions adjacent to the N-glycosylation site. Such differences may affect the size and flexibility of the unfolded N-glycosylation site, which leads to the differences in the efficiency of glycosylation.

SAA4 is a constitutive apolipoprotein of HDL. Its physiological function is virtually unknown. The plasma concentration of SAA4 varies between individuals and has no relationship with other major apolipoproteins [9]. We reported previously that SAA4 could be minimally induced in inflammatory conditions [14]. If it plays a role in inflammation, the glycosylation status of SAA4 may have functional implications.

In a previous study, we reported that SAA1 polymorphism influenced SAA concentrations (specifically A-SAA concentrations) [15]. One of the reasons for this may be that SAA polymorphism affects the affinity of SAA to HDL followed by changes in plasma clearance, which was suggested by our experiments using recombinant SAA1 isotypes [16, 17]. The present study showed that polymorphism affecting glycosylation did not influence SAA4 concentration, suggesting that glycosylation may not play an active role in SAA4 metabolism at a level sufficient to affect plasma concentration. However, it may be worth investigating the interaction between SAA4 and HDl, focusing on SAA4 polymorphism and its related glycosylation status. It may be a trigger that can provide a hint on SAA4 function.

In conclusion, glycosylation of SAA4 was revealed to be influenced by polymorphism. Its biological significance, together with the biological function of SAA4, needs to be further investigated.
